# CANNABIS USE PERCEPTIONS, PATTERNS AND SYMPTOM MANAGEMENT IN OLDER VS. MIDDLE AGE AFTER A CANCER DIAGNOSIS

**DOI:** 10.1093/geroni/igac059.2840

**Published:** 2022-12-20

**Authors:** Margaret Fahey, Erin McClure

**Affiliations:** Medical University of South Carolina, Charleston, South Carolina, United States; Medical University of South Carolina, Charleston, South Carolina, United States

## Abstract

Prevalence of cannabis among U.S. older adults (> 65 years) is increasing and is also common among cancer patients of all ages. Less is known how cannabis beliefs and use differ in older vs. middle-age after cancer diagnosis. This NCI-funded P30 administrative supplement included a cross-sectional survey in a state with illegal access to cannabis. Participants (Nf1,038) had past cancer diagnosis (< 2 years). Comparisons were run between older (> 65 years; n=509) and middle-aged adults (45–64; n=378) relating to cannabis perceptions, patterns of use, and symptom management (among those endorsing use) with chi-square and t-test analyses. There were no age-group differences in prevalence of ever or current use, nor likelihood of discussing cannabis with provider. Use after diagnosis was less likely in older vs. middle-age (21% vs. 33%). Of those using after diagnosis, there were no age-group differences in reasons for use, but older adults reported less neuropathy relief and less digestive improvement from cannabis. When asked about potential benefits of cannabis, older adults were less likely to expect relief from neuropathy and nausea, increased appetite, decreased medication use, cancer cure, and treatment of another condition and more likely to expect improved sleep. When endorsing potential risks, older adults were more likely to report addiction and increased use of other substances and less likely to report legal reasons, job loss, and negative reactions. Findings indicate unique expectations and symptom management in older age, highlighting importance of oncology providers to discuss cannabis with this age group as rates rise.

